# Genetic gain and inbreeding from simulation of different genomic mating schemes for pig improvement

**DOI:** 10.1186/s40104-023-00872-x

**Published:** 2023-06-13

**Authors:** Fuping Zhao, Pengfei Zhang, Xiaoqing Wang, Deniz Akdemir, Dorian Garrick, Jun He, Lixian Wang

**Affiliations:** 1 Key Laboratory of Animal Genetics, Breeding and Reproduction (Poultry) of Ministry of Agriculture and Rural Affairs, Institute of Animal Science, Chinese Academy of Agricultural Sciences, Beijing, 100193 China; 2 Center for Blood and Marrow Transplant Research, Minneapolis, MN USA; 3 AL Rae Centre for Genetics and Breeding, Massey University, Hamilton, 3240 New Zealand; 4 College of Animal Science and Biotechnology, Hunnan Agricultural University, Changsha, 410128 China

**Keywords:** Genetic gain, Genomic mating, Genomic selection, Inbreeding, Pig

## Abstract

**Background:**

Genomic selection involves choosing as parents those elite individuals with the higher genomic estimated breeding values (GEBV) to accelerate the speed of genetic improvement in domestic animals. But after multi-generation selection, the rate of inbreeding and the occurrence of homozygous harmful alleles might increase, which would reduce performance and genetic diversity. To mitigate the above problems, we can utilize genomic mating (GM) based upon optimal mate allocation to construct the best genotypic combinations in the next generation. In this study, we used stochastic simulation to investigate the impact of various factors on the efficiencies of GM to optimize pairing combinations after genomic selection of candidates in a pig population. These factors included: the algorithm used to derive inbreeding coefficients; the trait heritability (0.1, 0.3 or 0.5); the kind of GM scheme (focused average GEBV or inbreeding); the approach for computing the genomic relationship matrix (by SNP or runs of homozygosity (ROH)). The outcomes were compared to three traditional mating schemes (random, positive assortative or negative assortative matings). In addition, the performance of the GM approach was tested on real datasets obtained from a Large White pig breeding population.

**Results:**

Genomic mating outperforms other approaches in limiting the inbreeding accumulation for the same expected genetic gain. The use of ROH-based genealogical relatedness in GM achieved faster genetic gains than using relatedness based on individual SNPs. The G_ROH_-based GM schemes with the maximum genetic gain resulted in 0.9%–2.6% higher rates of genetic gain Δ*G*, and 13%–83.3% lower Δ*F* than positive assortative mating regardless of heritability. The rates of inbreeding were always the fastest with positive assortative mating. Results from a purebred Large White pig population, confirmed that GM with ROH-based GRM was more efficient than traditional mating schemes.

**Conclusion:**

Compared with traditional mating schemes, genomic mating can not only achieve sustainable genetic progress but also effectively control the rates of inbreeding accumulation in the population. Our findings demonstrated that breeders should consider using genomic mating for genetic improvement of pigs.

**Supplementary Information:**

The online version contains supplementary material available at 10.1186/s40104-023-00872-x.

## Background

Animal breeding methods have changed dramatically over the past one hundred year [1]. One of the revolutionary changes has been the joint use of phenotypic and pedigree data to estimate breeding values through best linear unbiased prediction (BLUP) [2]. Based on the resultant predictions of genetic merit, selection has substantially improved animal production levels [3, 4]. At the beginning of the twenty-first century, the application of genomic selection (GS) technology once again innovated animal breeding methods [5, 6]. GS is based on ranking candidates using genomic estimated breeding values (GEBV) which are obtained using various BLUP approaches but with the addition of genotypes. With genomic information, GS improves selection accuracy at young ages which facilitates early selection or increased selection differentials and in some breeding programs can also shorten the generation interval [7]. Collectively, GS provides more accurate prediction of Mendelian sampling effects at young ages and can lead to improved genetic gains [8].

The long-term goal of selection should be to achieve sustainable genetic gain while population genetic diversity by restricting the rate of increase of inbreeding [9]. Reduced inbreeding reduces the probability of generating offspring that are homozygous for harmful genes and reduces the loss of low frequency alleles [10]. Optimal mating strategies generally balance the rate of genetic gain with the accumulation of inbreeding. In the late twentieth century, the method of optimal mating using pedigree relationships was put forward [11, 12]. While seeking to maximize genetic gain, these methods limit inbreeding by restricting matings between closely related animals, such as can be achieved using optimal contribution selection (OCS) [13, 14]. OCS can provide sustainable and long-term genetic gain from selection by maximizing the weighted genetic value of parents while simultaneously limiting the genetic relationship between them using the pedigree data of selection candidates.

OCS can be implemented using a variety of algorithms or methods. Methods implementing OCS theory have been proposed based on minimum coancestry or minimizing the covariance between ancestral contributions. These approaches tend to disperse the contribution of individuals in the breeding population and tend to increase the number of ancestors represented in each offspring. This brings the ancestors closer to the exact threshold linear relationship and reduces the inbreeding rate [15, 16]. Another option to optimize mating is to maximize a weighted index including descendant genetic merit and descendant inbreeding [17]. Optimizing that simple index with general-purpose meta heuristics, such as a differential evolution algorithm [18], allows one to comfortably accommodate alternative or additional objectives, thus trading the optimality of solutions for flexibility. Kinghorn [19, 20] used that algorithm to transform the problem of calculating the contribution rate to identify optimal mating combinations. The approach involved two parts: (i) a mate selection index, and (ii) a mate selection algorithm to be used to find the mating set which maximizes the response in the index. Such strategies have been referred to as look ahead mate selection schemes, as they involve predicting the outcome of alternative mate selections by considering the attributes of the offspring, and they can account for within-cross variance [21].

With the widespread adoption of genotyping for genome-wide single nucleotide polymorphisms (SNP), the realized genealogical relatedness can be derived, and collected into matrix of genomic relationships (GRM) [22]. Since GRM better reflects the actual Mendelian genetic sampling than the numerator relationship matrix (NRM), it is more accurate for prediction. In 2016, Akdemir et al. [23] proposed an approach known as genomic mating (GM) to obtain the best mating combination of parents for the next generation. The first application of GM was based on the whole population for breed conservation [23]. GM not only uses the genomic information that is the basis for genomic selection but also includes information on the complementation of parents to be mated. It determines which genotypes should be combined to obtain high performing offspring in the subsequent generation. Therefore, GM can control the population inbreeding level while enabling long-term and sustainable genetic gains.

In traditional breeding schemes, selecting individuals with genetically or phenotypically similar characteristics to mate is known as positive assortative mating. That mating scheme can achieve maximum short-term genetic gains but at the expense of increased rates of inbreeding. The opposite approach is negative assortative mating where selected animals are chosen as mating pairs when they exhibit dissimilar genetic or phenotypic characteristics. In this study, we investigate impacts of different factors on genomic mating in simulated and actual purebred pig populations. Based on the demographic history of the pig population, we simulated traits with different heritabilities. After selecting the best individuals as identified by the highest GEBV rankings, genomic mating is used to optimize mate allocation of those selected animals. Under different mating schemes, the average genetic gain and inbreeding coefficients in the offspring population were compared. There were three aims to this study: (1) evaluate whether implementing GM after GS can obtain the maximum genetic gain while effectively controlling the inbreeding accumulation compared with other traditional mating schemes, (2) explore the effects of using a GRM constructed from SNP genotypes compared to a GRM based on runs of homozygosity (ROH) of SNP genotypes, and (3) validate the application using real data from a purebred pig population.

## Materials and methods

### Simulation data

#### Simulation of the foundation and initial reference populations

Based on the demographic history of pigs, the QMSim [24] software was used to simulate pedigree and genomic data with random mating and discrete generations to reconstruct an ancestral foundation population. The parameters and breeding structure followed a previous study [25]. The simulation process was divided into two steps. The first step was to create realistic levels of linkage disequilibrium (LD) from an ancestral foundation population and to establish mutation-drift equilibrium using a mutation rate of 2.5 × 10^–5^. The population size each generation was 2,000 individuals, consisting of 1,000 males and 1,000 females. The sire: dam mating ratio was 1:1 and the number of offspring per mating was 10 with an equal sex ratio. After 1,000 generations of random mating, the population size was gradually reduced to include only 400 individuals for the next 1,000 generations. In a second step, we selected 30 males and 900 females from the last generation of the ancestral population to represent modern founders. Each male mated 30 females randomly. The litter size was 10 with an equal sex ratio. The resultant offspring and their subsequent generation of 9,000 offspring represented the reference population of animals with pedigree, performance and genomic information to begin genomic prediction.

The simulated genome consisted of 18 pairs of chromosomes of 100 cM each. The number of SNP markers on each chromosome was 1,700 so the total number of SNPs was 30,600. Both SNPs and quantitative trait loci (QTL) were biallelic and evenly distributed on all the chromosomes. The detailed genome parameters simulated are listed in Table 1.

**Table 1:** The parameters of genomic information in simulation population

Genomic parameters	Setting
Number of chromosomes	18
Chromosome length, cM	100
Number of marker loci per chromosome	1,700
Number of QTL loci per chromosome	17
Marker positions	Random
QTL positions	Random
The marker mutation rate in the historical population	2.5 × 10^–5^
The QTL mutation rate in the historical population	2.5 × 10^–5^

Genetic and phenotypic values were simulated for three traits for every individual. The additive genetic effect of QTLs was sampled from a gamma distribution with a shape parameter $\alpha$ = 0.4. The traits all had a phenotypic variance of 1,000 but the heritability differed by trait and was 0.1, 0.3 or 0.5. The effect of QTLs was considered to explain 100% of the genetic variance. The genetic variance was $\sigma_{g}^{2} = h^{2} \times \sigma_{p}^{2}$, and the environmental variance was $\sigma_{e}^{2} = \sigma_{p}^{2} - \sigma_{g}^{2}$.

#### Simulation of offspring genotypes

The simulated offspring will have inherited alleles at each locus following the principles of Mendelian inheritance. When QMSim was used to simulate the base population data (generation 0), the haplotypes of the parents were defined, each locus was represented by two alleles (1 and 2), and the first allele was from the sire whereas the second allele was from the dam. Thus, the genotype of the offspring was simulated from the genotypes of the parents. The specific process was as follows [26]: (1) For the first locus: a random number *μ* was generated from the uniformly distribution [0, 1]. If *μ* < 0.5, the first allele at the first locus of an individual was inherited from the paternal chromosome of the sire, if *μ* > 0.5, the first allele was inherited from the first locus on the sire’s maternal chromosome. (2) For the *i*^th^ (*i* = 2, …, *N*) locus, the recombination rate between the two adjacent loci was calculated according to the Haldane mapping function [27], using the following equation:

$$r=\frac{1}{2} \left(1-{e}^{-2c}\right)$$

where *c* is the genetic distance (in Morgan) between the *i*^th^ locus and the (*i *− 1)^th^ locus. Then a uniformly distributed [0, 1] random number *μ* was sampled, and if *μ* > *r*, no recombination occurred, and the first allele at the *i*^th^ locus of the individual came from the *i*^th^ locus on the same chromosome of the sire that contributed the allele for the (*i *− 1)^th^ locus. If *μ* < *r*, recombination occurred, and the first allele at the *i*^th^ locus of the individual came from the *i*^th^ locus on the other chromosome of the sire. (3) The remaining markers followed this process for simulating inheritance of the paternal alleles. (4) The same process was repeated to sample the maternally inherited alleles of the individual.

#### Simulation of true breeding and phenotypic values of offspring individuals

The true breeding values of individuals were generated according to the following equation:

$${\text{g}}_{i}=\frac{1}{2} {\text{g}}_{s} + \frac{1}{2} {\text{g}}_{d} + {w}_{i}$$

where *g*_s_ is the breeding value of the sire, *g*_d_ is the breeding value of the dam, and *w*_*i*_ is the Mendelian sampling term of individual *i*, which is the summation of Mendelian sampling errors of sire and dam using the following equation: $w_{i} = w_{s} + w_{{d}}$, where *w*_s_ is the Mendelian sampling error of the sire, and *w*_*d*_ is the Mendelian sampling error of the dam. Hence, *w*_*i*_ follows the *N* (0, $\sigma_{w}^{2}$) distribution, which is $\sigma_{w}^{2} = \frac{1}{4}(1 - f_{s} )\sigma_{a}^{2} + \frac{1}{4}(1 - f_{d} )\sigma_{a}^{2}$, where $f_{{\text{s}}}$ and $f_{d}$ are the inbreeding coefficients of individual *i*’s sire and dam, respectively, and $\sigma_{a}^{2}$ is the genetic variance of the trait in the base population.

The phenotypic values of individuals were simulated according to the following model:

$${y}_{i} = \mu + {\text{g}}_{i} + {e}_{i}$$

where $y_{i}$ is the phenotypic value of individual *i*, *μ* is the population mean, *g*_*i*_ is the random additive genetic effect (true breeding value) of individual *i*, and *e*_*i*_ is the random residual of individual *i* which follows *N* (0, $\sigma_{{\text{e}}}^{2}$) in every generation.

#### GEBV estimation and individual selection

The marker effects were estimated by BayesB using the R package BGLR [28] and based on the two generations of pedigree, phenotypic and genomic information that collectively comprised the reference population. The number of MCMC samples, burn-in and thinning were 20,000, 1,000 and 20, respectively. The males and females in the most recent generation were sorted according to GEBV, and the top 30 sires and top 900 dams were selected as breeding individuals for parenting the next generation. This process was repeated in each generation. However, the reference population used for genomic prediction only ever consisted of the individuals in the previous two generations, which were used to re-estimate marker effects based on repeatedly fitting a BayesB model [28].

#### Genomic mating schemes

In this study, both genetic gain and inbreeding coefficients were taken into account in defining the objective function for GM. The specific formulas for quantifying genetic gain and inbreeding coefficients were as follows:

$$Inbreeding\left({\varvec{P}}\right)={\boldsymbol{1}}_{{N}_{C}}^{\mathrm{^{\prime}}} \left({\varvec{P}}{\varvec{G}\varvec{P}}^{^{\prime}} + {{\varvec{D}}}\right)\boldsymbol{1}_{{N}_{C}}$$

$$Gain({\varvec{P}})={1}_{{N}_{C}}^{\mathrm{^{\prime}}}\,{\varvec{P}}{\varvec{G}}{\varvec{M}}{\varvec{a}}$$

where ***P*** is the incidence matrix reflecting mating pairs of order *Nc* × *N*, where *Nc* is the number of offspring and* N* is the number of parents; ***G*** is a genomic relationship matrix; ***D*** is the Mendelian sampling dispersion; ***M*** is the genotype matrix; ***a*** is the vector of BayesB estimated marker effects.

In this study, two formulations of a GRM were constructed for the calculation of the inbreeding coefficients from genomic mating. Namely: (1) A GRM calculated using the formula in VanRaden [22] by A.mat function in the R package rrBLUP [29]. This function employed the equation: ${\varvec{G}}=\frac{{\varvec{M}}{{\varvec{M}}}^{^{\prime}}}{2{\sum }_{i}^{k}{p}_{i}(1-{p}_{i})}$, where ***M*** is the genotypes and *p*_*k*_ is the minor allele frequency at marker *k*. (2) A GRM based on the ROH relationship matrix which was 2 times the segment-based kinship matrix computed using the segIBD function in the R package optisel [30]. The haplotypes were constructed in advance of the calculation of the segment-based kinship matrix.

To obtain the optimal mate allocation, the R package TrainSel [31] was executed in GM schemes. Parameter settings were below: the number of sires, dam and mating combinations were set to 30, 900 and 900, respectively; the population size in the genetic algorithm parameters was set to 200; the number of iterations was set to 800; and the remaining parameters were set by default.

#### Traditional mating schemes

There were three traditional mating schemes as below:

1. Random mating. The 30 sires and 900 dams selected by GS were randomly mated, with no restriction on mating among siblings.
2. Positive assortative mating. The 30 sires and the 900 dams selected by GS were sorted in the order of GEBV, then the highest ranking sire was mated with the 30^th^ highest ranking dams, and so on, until the rank 30 sire was mated to the 871–900^th^ ranking dams with no restriction on sibling mating.
3. Negative assortative mating. The 30 sires selected by GS were sorted in the order of GEBV from low to high, and the 900 dams were sorted in the order of GEBV from high to low. The highest ranking sire was mated to the 30 lowest ranking of the 900 dams, and so on, until the rank 30 sire was mated to the 30 highest ranking of the 900 dams, with no restriction on siblings mating.

#### Evaluation criteria

Three factors were used to evaluate the effects of mating schemes including the rate of genetic gain and the rate of inbreeding in the offspring. The detailed information is below:

1. The rate of genetic gain was calculated using the following equation: $\Delta G = \overline{a}_{u} - \overline{a}_{u - 1}$ where $\overline{a}$ is the average GEBV, and *u* is the generation number.
2. Two measures of inbreeding were used. One was the pedigree-based inbreeding coefficient (*F*_*PED*_) proposed by Wright [32], namely $F_{PED} = \sum {(\frac{1}{2})^{N} \left( {1 + F_{A} } \right)}$, where *N* is the number of related pathway chains from the individual's sire to the common ancestor and *F*_*A*_ is the inbreeding coefficient of the common ancestor *A*. The other was the estimated inbreeding coefficient (*F*_*GRM*_) based on SNPs:$F_{GRM} = \sum\nolimits_{i = 1}^{m} {([x_{i} - E(x_{i} )]^{2} /[2p_{i} (1 - p_{i} ) - 1])} /m$, where *m* is the number of SNPs, *p*_*i*_ is the minor allele frequency, and *x*_*i*_ is the copy number of the *i*^th^ SNP. Inbreeding rate, Δ*F*, was calculated as 1 − *e*^*β*^, where *β* is the slope of the linear regression of ln (1 − *F*_*u*_) on *u* and *F*_*u*_ is the mean inbreeding coefficient for animals born at the *u*^th^ generation, as used in Nirea et al. [33].

#### Breeding Schemes

**Fig. 1:**
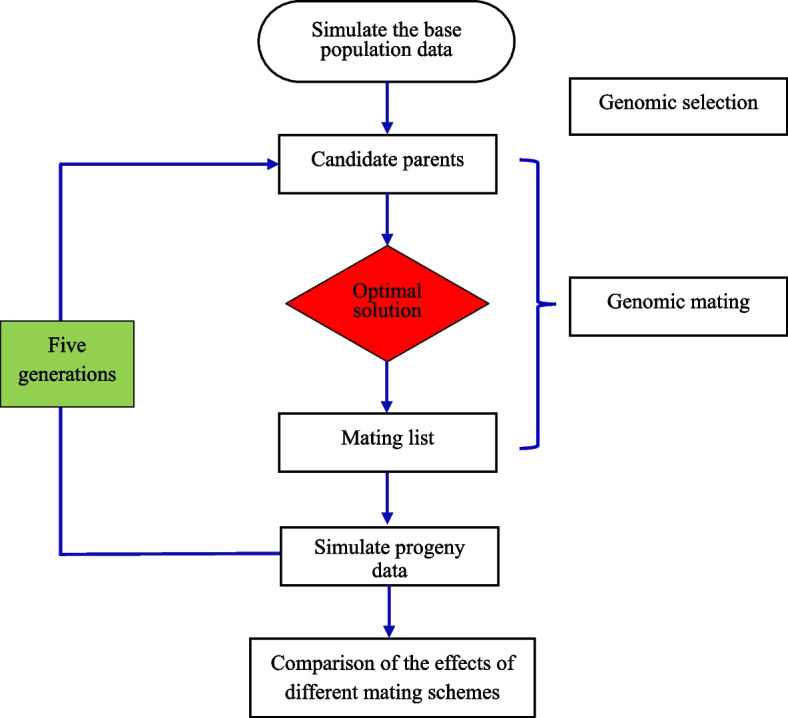
Technical schematic of the simulation study

This study makes the following assumptions: (1) There is no overlap between generations; (2) Environmental variation is homogeneous across generations; (3) Every individual has a phenotypic value for all 3 traits; (4) The litter size is 10. The specific process is shown in Fig. 1. Three traditional mating schemes (random mating, positive assortative mating and negative assortative mating) or four genomic mating schemes focused on either the average GEBV or inbreeding via the use of GRM constructed by SNP or ROH were used to allocate mates after genomic selection. Each breeding scheme was continued for five generations, and the average GEBV, rate of genetic gain and average inbreeding coefficient in each generation for each different scheme were calculated. There were five replicates. All the above simulation calculations were scripted in the R language and run on a Linux system.

### Empirical data

The performance of the genomic mating program was tested on a real dataset obtained from a herd of purebred Large White pig breeding population run by a commercial company in Shanghai city. Only the total number of piglets born was analysed and the data for that trait consisted of 16,326 records. The pedigree data contained 57,135 animals and was used in a repeatability animal model to estimate the EBV following Wang et al. [34]. Some 6,265 of the animals had been genotyped with a GeneSeek Porcine 50K array. After standard quality control, 43,465 autosomal SNPs were retained. Haplotypes were phased, and missing genotypes were imputed using Beagle software [35]. We used pre-corrected phenotypes, computed as the sum of the estimated breeding value (EBV) and the residual for estimating the SNP marker effects. Thirty-eight males and 307 females that had been genotyped were selected to be parents of the next generation, and their GEBVs was 0.016. The total number of their genotyped offspring was 504. The average number of paternal half- and full-sibs was 13.263 ± 14.06 with a range of 1–55, while the average number of dam’s offspring was 1.64 ± 1.00 with the range of 1–5. The average of GEBVs of their offspring was −0.086 and the average of half of the sum of their parents’ GEBVs was −0.09754.

## Results

### Determining criteria for genomic mating schemes

**Fig. 2:**
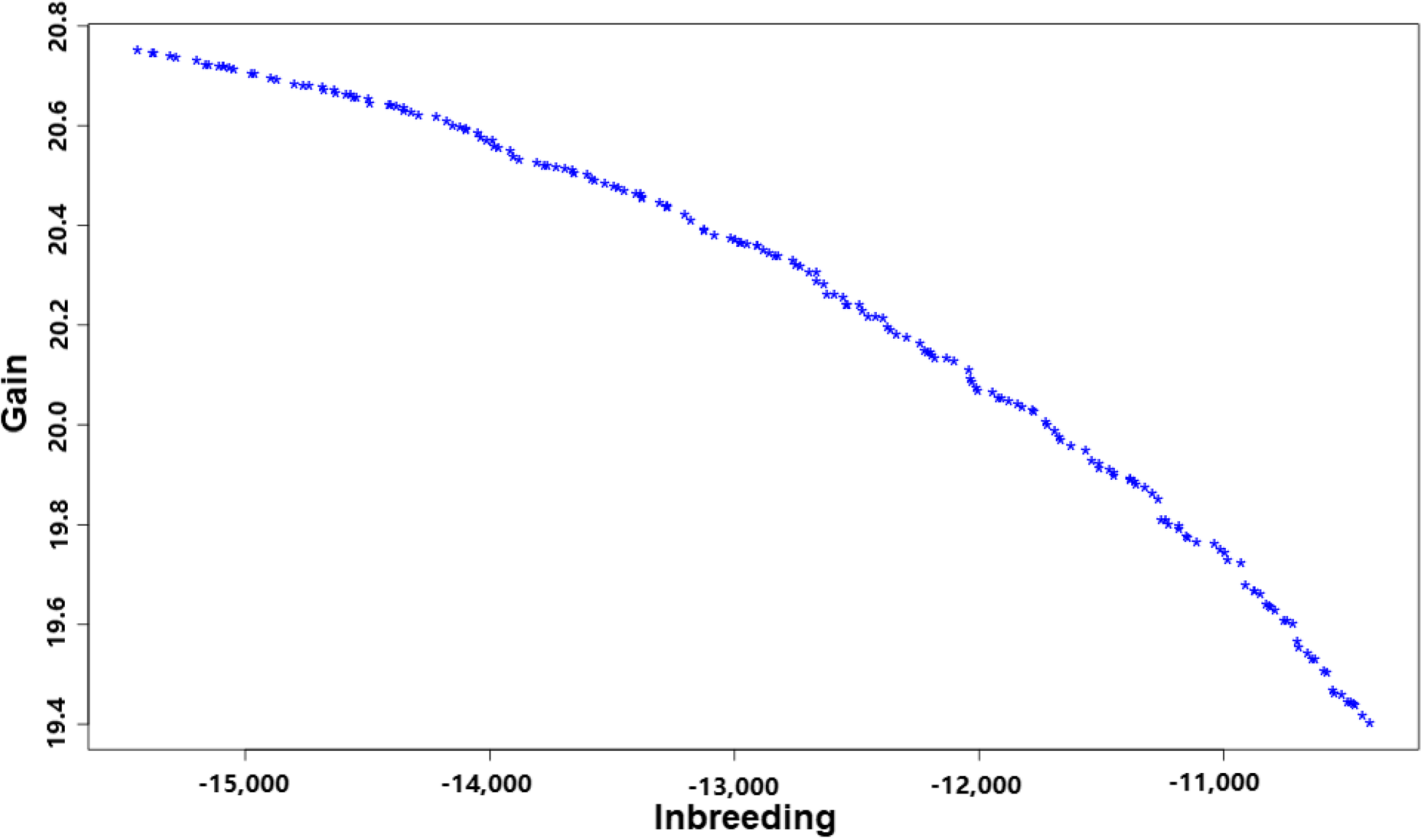
The optimal solution for genomic mating

Genetic gain and inbreeding are not independent but antagonistic since more intense selection will increase both genetic gain and inbreeding and thereby reduce effective population size. By balancing these two key performance indicators (KPI), GM will obtain a series of solutions, as shown in Fig. 2. Each point in the graph corresponds to the values of the two KPI for a set of mating combinations. Any of the points on the surface of the graph can be used as feasible mating schemes for obtaining the next generation. The optimal scheme needs to define the relative utility of gain vs. inbreeding. Different managers may have different utility functions and therefore choose different schemes as being optimal. As seen in Fig. 2, after applying GS to choose the parents, the highest point in the left indicated the genomic mating scheme with maximum genetic gain and maximum inbreeding, while the lowest point in the right suggests the genomic mating scheme with minimum genetic gain and minimum inbreeding. These two mating schemes generate the upper and low limit results. In this study, we selected the mating combinations of the solution with the maximum genetic gain or the minimum inbreeding as the optimal solution, according to the different mating combinations specified by these two KPI. In this study, we should note that the GM scheme focused on maximum genetic gain via the use of G or G_ROH_ was denoted by GM_G_Gain or GM_G_ROH__Gain, respectively, and the genomic mating scheme focused on the minimum inbreeding via the use of G or G_ROH_ was denoted by GM_G_Inb and GM_G_ROH__Inb, respectively.

### Results of different mating schemes with the different heritabilities

#### Heritability of 0.1

**Fig. 3:**
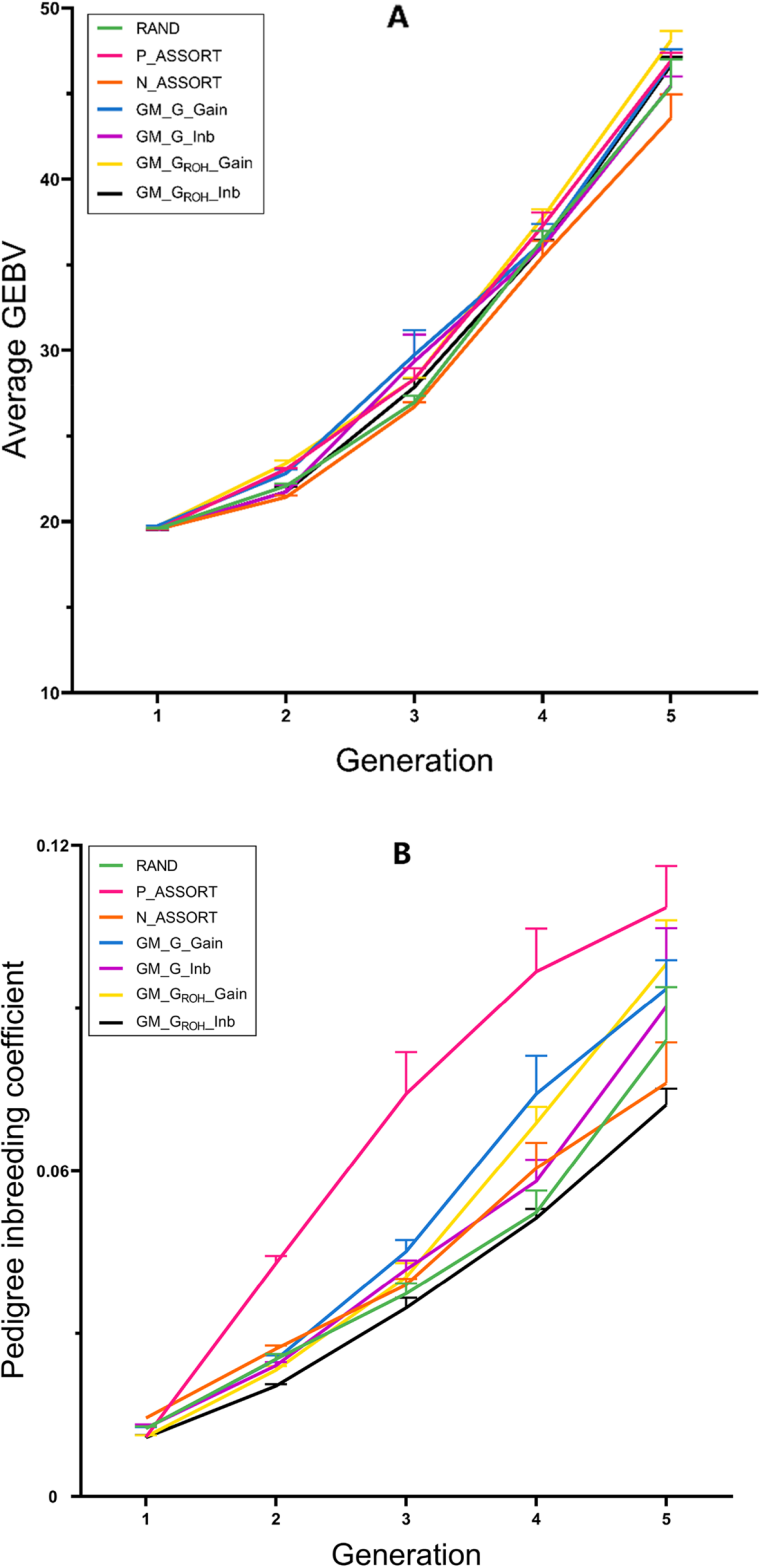
Genetic trend and pedigree inbreeding coefficient of seven different mating schemes after five generations of breeding at the heritability of 0.1

The genetic trend of different mating schemes at the heritability of 0.1 is shown in Fig. 3A. Comparing across scenarios, maximum gain (highest average GEBV) in the first generation (19.759) was via the use of G (GM_G_Gain), which was higher than the three traditional mating schemes (19.592, 19.559 and 19.556). But after five generations of breeding, the per generation increase in average GEBV of GM_G_ROH__Gain (9.296) was the highest, which was higher than negative assortative mating (8.387). GM_G_Gain (9.042), GM_G_Inb (8.768), GM_G_ROH__Gain and GM_G_ROH__Inb achieved 0.2% to 6.2% more Δ*G* than random mating (8.754) and 4.5% to 10.8% than negative assortative mating. Moreover, the Δ*G* of the GM_G_ROH__Gain was 2.7% and 2.8% higher than positive assortative mating and GM_G_Gain, respectively (Table 2).

**Table 2:** Average rate of genetic gain (Δ*G*) and average rate of inbreeding (Δ*F*) realized in simulation by generations 1 to 5 from seven different mating schemes at three heritabilities

*h*^2^	Mating scheme	Δ*G*	Δ*F*
0.1	RAND	8.754 ± 0.632	0.018 ± 0.005
	P_ASSORT	9.055 ± 0.187	0.026 ± 0.005
	N_ASSORT	8.387 ± 0.554	0.016 ± 0.004
	GM_G_Gain	9.042 ± 0.292	0.022 ± 0.004
	GM_G_Inb	8.768 ± 0.211	0.020 ± 0.007
	GM_G_ROH__Gain	9.296 ± 0.215	0.023 ± 0.004
	GM_G_ROH__Inb	9.001 ± 0.186	0.016 ± 0.002
0.3	RAND	16.694 ± 1.559	0.018 ± 0.003
	P_ASSORT	18.426 ± 1.401	0.044 ± 0.009
	N_ASSORT	17.527 ± 0.987	0.022 ± 0.003
	GM_G_Gain	18.362 ± 0.379	0.021 ± 0.004
	GM_G_Inb	17.146 ± 0.127	0.017 ± 0.003
	GM_G_ROH__Gain	18.601 ± 0.380	0.024 ± 0.005
	GM_G_ROH__Inb	17.937 ± 0.386	0.017 ± 0.004
0.5	RAND	25.377 ± 1.129	0.031 ± 0.006
	P_ASSORT	29.241 ± 1.069	0.065 ± 0.011
	N_ASSORT	24.123 ± 0.485	0.019 ± 0.002
	GM_G_Gain	28.698 ± 1.561	0.041 ± 0.009
	GM_G_Inb	26.986 ± 1.008	0.027 ± 0.003
	GM_G_ROH__Gain	29.901 ± 2.530	0.047 ± 0.020
	GM_G_ROH__Inb	26.281 ± 0.504	0.024 ± 0.006

Figure 3B shows the trend of the pedigree-based inbreeding coefficient (*F*_*PED*_) of different mating schemes at the heritability of 0.1. The *F*_*PED*_ of positive assortative mating showed a rapid upward trend, especially in the third generation, which was higher than other schemes. The *F*_*PED*_ of GM_G_Gain was lower than positive assortative mating in the second and third generations. The *F*_*PED*_ values of GM_G_ROH__Gain in 2–4 generations were significantly lower than those of positive assortative mating, but in the fifth generation, it was higher than GM_G_Gain. The *F*_*PED*_ of the GM_G_ROH__Inb was the lowest among all schemes. The Δ*F* of the four genomic mating schemes were 13%–62.5% lower than positive assortative mating, and the Δ*F* of GM_G_ROH__Inb was the same as that of negative assortative mating, which was 12.5% lower than random mating and 43.75% lower than GM_G_Gain. The Δ*F* of GM_G_Gain was 4.5% lower than GM_G_ROH__Gain (Table 2).

#### Heritability of 0.3

**Fig. 4:**
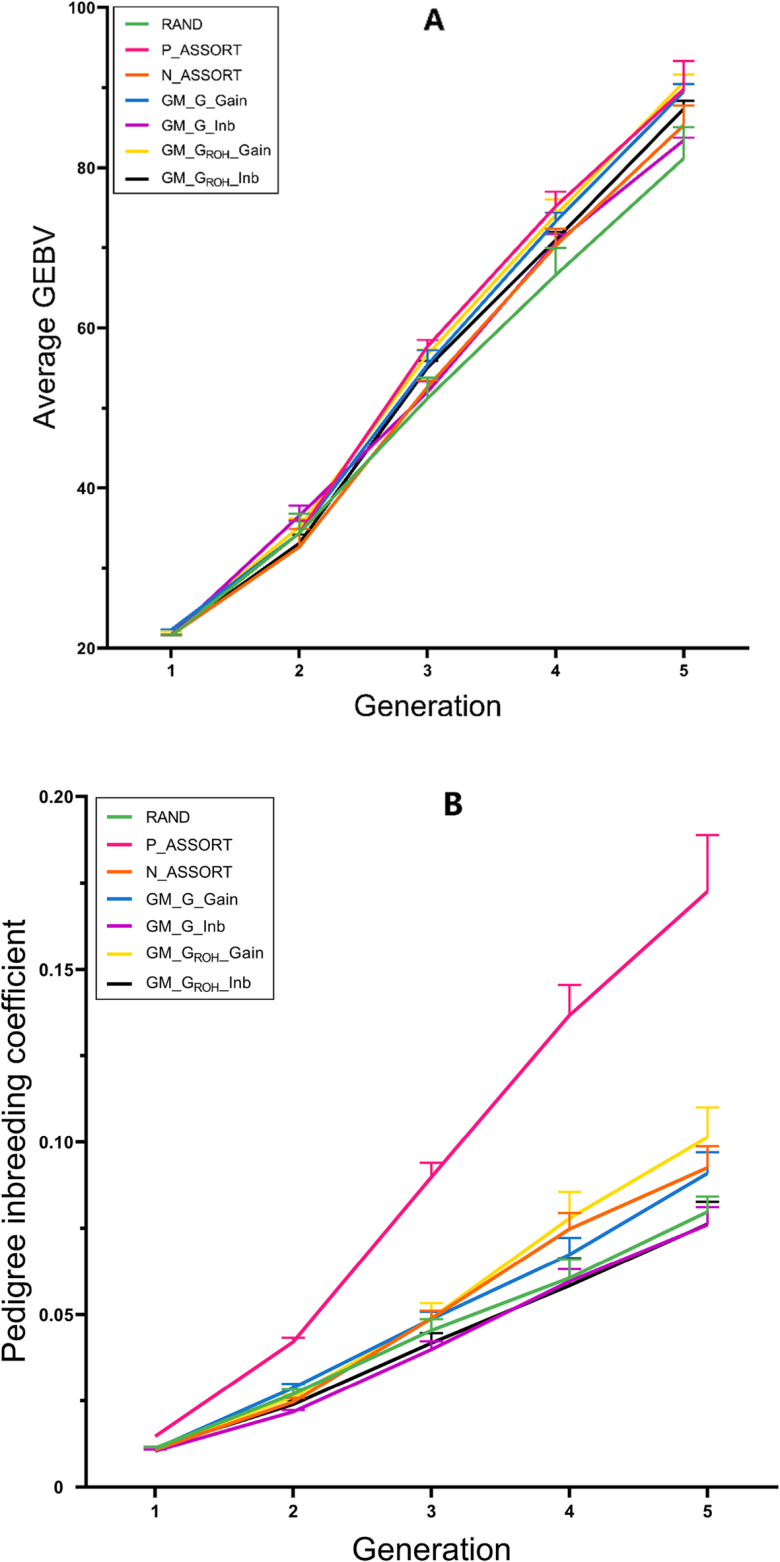
Genetic trend and pedigree inbreeding coefficient of seven different mating schemes after five generations of breeding at the heritability of 0.3

The genetic trend of different mating schemes at the heritability of 0.3 is displayed in Fig. 4A. In the first generation, the average GEBV of GM_G_Gain (22.305) was the biggest among all the mating schemes. From the second to the fifth generations, it was lower than positive assortative mating, but it was higher than random mating. GM_G_ROH__Inb (87.386) was higher than that of negative assortative mating (82.173) and random mating (83.339) in the fifth generation. The average GEBVs of GM_G_ROH__Gain from the second to the fifth generation had accelerated rapidly. In the fifth generation, it (90.710) was higher than random mating. The Δ*G* of the four genomic mating schemes was 2.7%–11.4% higher than random mating. The Δ*G* of GM_G_ROH__Gain (18.601) was the highest among all mating schemes, 1.0% higher than positive assortative mating, and 1.3% higher than GM_G_Gain (Table 2).

The trend of the pedigree-based inbreeding coefficient of different mating schemes at the heritability of 0.3 is shown in Fig. 4B. The *F*_*PED*_ of positive assortative mating showed a rapid upward trend and was extremely higher than other mating schemes in the five generations. The *F*_*PED*_ of GM_G_Inb and GM_G_ROH__Inb were lower than the other three traditional mating schemes. The *F*_*PED*_ of GM_G_Inb in the fourth generation was higher than that of GM_G_ROH__Inb and lower than that of GM_G_ROH__Inb in other generations. The *F*_*PED*_ of GM_G_ROH__Gain in the fourth generation exceeds that of GM_G_Gain, and in the fifth generation, it was higher than other mating schemes but far lower than positive assortative mating. The Δ*F* of the four genomic mating schemes was 83.3%–158.8% lower than positive assortative mating, among which GM_G_ROH__Inb and GM_G_Inb were 5.9% lower than random mating, and 29.4% lower than negative assortative mating. The Δ*F* of GM_G_ROH__Gain was 14.3% higher than GM_G_Gain, and the Δ*F* of GM_G_Inb was the same as that of GM_G_ROH__Inb (Table 2).

#### Heritability of 0.5

**Fig. 5:**
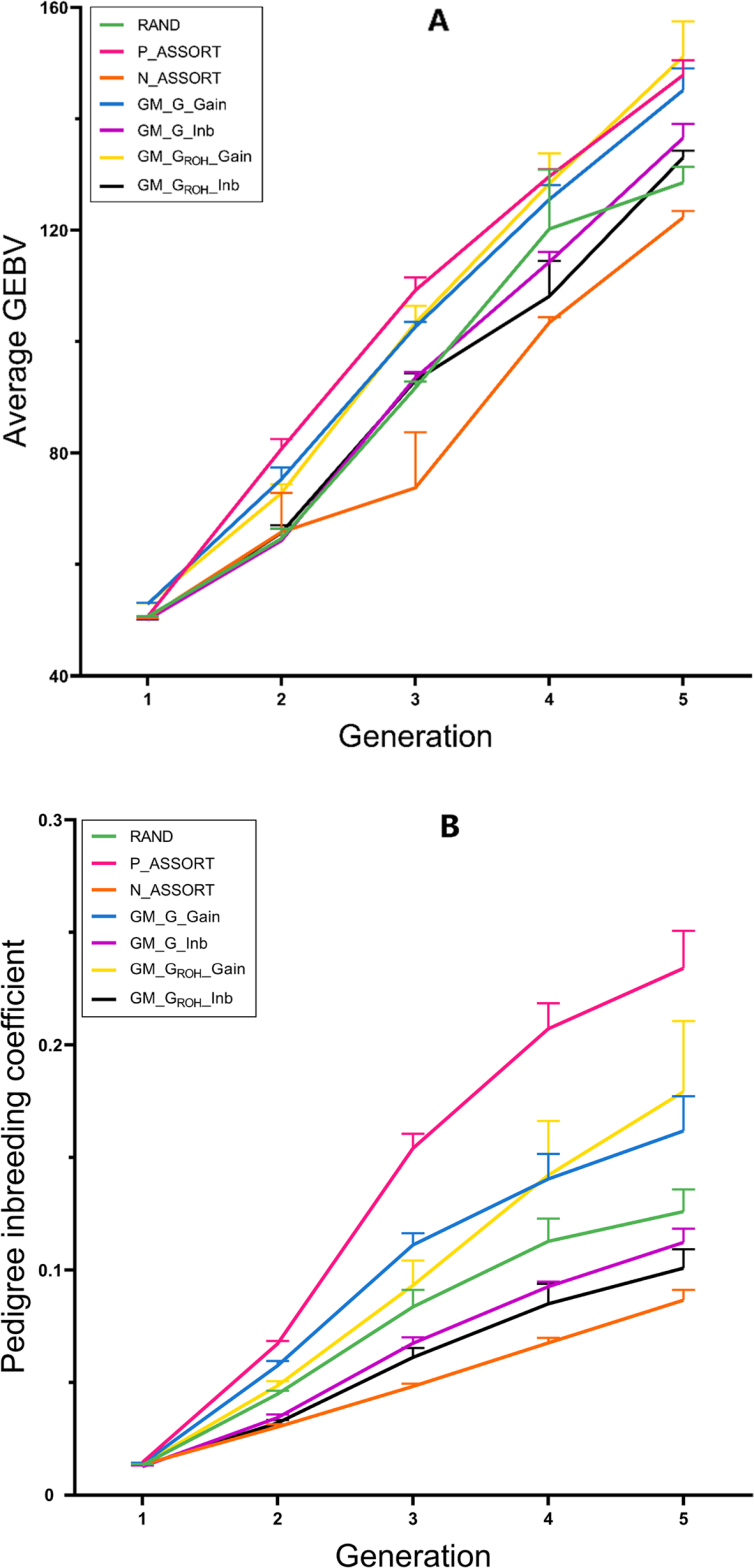
Genetic trend and pedigree inbreeding coefficient of seven different mating schemes after five generations of breeding at the heritability of 0.5

The genetic trend of different mating schemes at the heritability of 0.5 is depicted in Fig. 5A. The average GEBV of the GM_G_Gain (52.933) and GM_G_ROH__Gain (52.951) in the first generation was higher than those of the three traditional mating schemes, and the average GEBV of the two schemes increased simultaneously. In the fifth generation, the GM_G_ROH__Gain (151.161) had the highest average GEBV, which was higher than random mating (128.539), negative assortative mating (122.271), and GM_G_ROH__Inb (133.062). The Δ*G* of the four mating schemes in genomic mating was higher than those of random mating and negative assortative mating, 3.6%–17.8% higher than random mating, and 9%–24% higher than negative assortative mating. GM_G_ROH__Gain was 2.3% higher than positive assortative mating and 4.2% higher than GM_G_Gain. The Δ*G* of GM_G_Inb was 2.7% higher than GM_G_ROH__Inb (Table 2).

The trend of the pedigree-based inbreeding coefficient of different mating schemes at the heritability of 0.5 is illustrated in Fig. 5B. The *F*_*PED*_ of positive assortative mating in five generations was higher than those of random mating, negative assortative mating, GM_G_ROH__Inb, and GM_G_Inb, and was higher than that of GM_G_Gain and GM_G_ROH__Gain in second to fifth generation. The *F*_*PED*_ of GM_G_Gain increased faster in the second and third generations, but the *F*_*PED*_ increased slowly in the fourth and fifth generations. GM_G_Inb had the lowest *F*_*PED*_ in the first generation, higher than GM_G_ROH__Inb and negative assortative mating in second to fifth generations, but much lower than random mating. The Δ*F* of the four genomic mating schemes was 38.3%–170.8% lower than positive assortative mating, in which GM_G_ROH__Inb and GM_G_Inb were 14.8%–29.2% lower than random mating. The Δ*F* of GM_G_ROH__Gain was 14.6% higher than that of GM_G_Gain, and GM_G_Inb was 12.5% higher than GM_G_ROH__Inb (Table 2).

### Genomic mating in a real dataset of purebred pigs

To validate the results of simulation study, seven mating schemes were carried out by simulation based on real data. Moreover, the number of offspring was set to be 16 according to the real mean of the Large White pig population. So one male mated approximately 8.07 females in simulation study. As seen in Table 3, the largest average GEBV was showed in GM_G_ROH__Gain while the smallest one was showed in negative assortative mating among seven mating schemes. GM_G_Inb had the smallest average *F*_*PED*_ while positive assortative mating had the largest among seven mating schemes. The magnitude of average GEBV and average *F*_*PED*_ in random mating were between the positive assortative mating and negative assortative mating. G_ROH_-based genomic mating scheme had the largest average GEBV and average *F*_*PED*_ in four genomic mating schemes. Results indicated that genomic information was much more important in the analysis of real data than that of simulated data.

**Table 3:** Average GEBV and *F*_*PED*_ of seven different mating schemes in purebred Large White pig population

Mating scheme	MeanGEBV	Mean*F*_*PED*_
RAND	0.040 ± 0.001	0.023 ± 0.001
P_ASSORT	0.049 ± 0.047	0.032 ± 0.000
N_ASSORT	0.035 ± 0.001	0.021 ± 0.000
GM_G_Gain	0.310 ± 0.042	0.023 ± 0.001
GM_G_Inb	0.047 ± 0.003	0.018 ± 0.002
GM_G_ROH__Gain	0.333 ± 0.016	0.023 ± 0.002
GM_G_ROH__Inb	0.057 ± 0.001	0.021 ± 0.002

## Discussion

In this study, after selecting candidates based on GEBVs, GM utilized genomic information to get the optimal solution for the mating list. GS had been employed in the past decade in pigs, and it can significantly improve the genetic gain, but the use of GS for multiple generations would lead to increase of inbreeding level and decrease the genetic diversity. Therefore, it is necessary to control the rate of increase in the inbreeding level of the population.

Positive assortative mating involves mating between individuals with higher GEBVs, and in our study it produced the largest Δ*F* at all heritabilities. However, it will make the population obtain homozygosity more quickly after consecutive generations of selection. Hence, this scheme should usually be avoided in reality.

In this study, the genetic relationship of individuals in GM was constructed by SNP or ROH. Compared to SNPs, ROH can allow for lengths of the genomic regions shared between individuals, which can track the way that the alleles inherited from parents to offspring more accurately [36]. So, ROH-based genealogical relatedness is more accurate than relatedness based on single-SNP statistics. Our results also showed that the genetic gain of GM_G_ROH__Gain was higher than that of GM_G_Gain and the Δ*F* of GM_G_ROH__Inb was lower than those of GM_G_Inb at all heritabilities. Luan et al. [37] reported that G_ROH_ can generate more accurate GEBV compared to genomic relationship matrix by simulation. Moreover, some of *F*_GRM_ values would be negative, which was consistent with other studies [38–41]. Nevertheless, we didn’t observe any trends only using ROH-based inbreeding coefficients to assess the inbreeding trend in all mating schemes, especially for traditional mating schemes. This phenomenon was also exhibited in the analysis of real data. The possible explanation is G_ROH_ could not efficiently evaluate genomic mating with other relationship matrices or traditional mating systems. We finally used average *F*_*PED*_ of individuals to evaluate the population inbreeding level. There are two reasons: 1) *F*_*PED*_ value is the statistical expectation of the probable genomic proportion of identity by descent (IBD) [42]; 2) the complete pedigree information can be obtained in our simulation study.

GM needs estimating marker effects, and combine inbreeding coefficients and the estimated marker effects to determine which genotypes should be crossed to produce progenies [23, 43]. In the simulation study, the additive genetic effect of QTLs were sampled a gamma distribution with a shape parameter *α* = 0.4. There are several major effects related to target traits. The SNP effects across the whole genome under different heritabilities were shown in Fig. S1–3. We directly employed BayesB to estimate the SNP effects since BayesB is expected to perform well in such scenarios with major loci [44]. However, such architectures are rare in practice. This suggests that a method like BayesB risks being over-optimistic about what prediction accuracies can be achieved. The performance of different methods to estimate the SNP effects will be explore in future.

There are numerous algorithms to solve the GM optimization problem [43]. In this study, we used a hybrid heuristic optimization algorithm that combines genetic algorithm with simulated annealing for solving combinatorial optimization problems. This algorithm was implemented by the R package TrainSel [31]. This package can be used to select multiple ordered or unordered samples from lists of candidates. It has mostly been used for the selection of training populations [31]. In this study, we extended it to mate allocation. Using the R package TrainSel for genomic mating, there are several parameters requiring consideration including the number of sires and dams, mating combinations, number of SNPs, and in relation to the optimization algorithm, the population size in genetic algorithm parameters, and the number of iterations. All of them impact on the computing speed, while the population size and the number of iterations in genetic algorithm parameters directly affect whether the ideal results can be obtained. In this study, 30 sires and 900 dams were selected for each generation. Thus, there were 27,000 possible mating combinations, which required considerable computing effort. Through preliminary experiments, we found that the ideal results can be obtained when the population size in the genetic algorithm parameters in TrainSel was set to 200 and the number of iterations was set to more than 500. In this study, the population size of offspring was 9,000, the population size was set to 200 and the number of iterations was set to 800 in the parameters of the genetic algorithm, which will greatly increase the amount of calculation. In this study, 1,700 markers on each chromosome were simulated with a total of 30,600 markers. It took about 15 h each run on a 40 core 2.40 GHz Intel (R) Xeon (R) gold 6,148 CPU and 768 GB memory Linux server.

In this study, a series of optimal mating combinations can be obtained from TrainSel based on genomic information. Although the optimal set of mating combinations was not unique, all of them were better than other non-genomic mating schemes. The genetic algorithm using in TrainSel is still significantly different from others such as optimal genetic contribution selection [45–47]. The optimal genetic contribution selection only gives the proportion of the genetic contribution of the candidate parents to the offspring and does not give specific mating combinations. But genomic mating shifts the focus to mate selection by constructing mating matrices. The parental contribution ratio can be calculated through genomic mating, but not from the optimal genetic contribution selection.

In the empirical data analysis, the average of GEBV in the real offspring was smaller than those of all these mating data by simulation. There are two factors influencing the results: sire: dam mating ratio and the number of offspring per mate allocation. In the simulation analysis based on the real data, the average of the sire: dam mating ratio was about 8.07 and the number of offspring per mating allocate was 16, however they varied in real population. Both of them can directly affect the independence of genetic contributions of ancestors [48].

Relative to GS, GM also uses the estimated marker effects and the genetic information to decide which genotypes should be crossed to obtain the next breeding population. In the current study, we only focused on the additive genetic effects of a single trait in GM in the purebred breed. In addition to being used to optimize the mating scheme, GM can also be used to estimate crossbred animals, predict the probability of occurrence of high-yielding or low-yielding individuals, etc. [49]. At present, the implementation of GM is still in the preliminary stage, and there are still many practical problems worthy of further exploration.

## Conclusion

In this study, we used simulation to investigate the effects of different genomic mating schemes in pig breeding. Our simulation study shows that implementing genomic mating after genomic selection is more beneficial than genomic selection followed by traditional mating systems in pig breeding programs. The use of ROH-based genealogical relatedness in genomic mating can obtain the optimal solution with the maximum genetic gain. Genomic mating not only achieves sustainable genetic progress but also control rates of inbreeding. The real data results further validated the simulate study. Through the optimization and tradeoff of genetic gain and inbreeding, a series of optimal solutions are calculated for breeders to choose according to the real condition. Our findings contribute to understanding the effect of using genomic mating in pig genetic improvement.

## Supplementary Information

**Additional file 1: Fig. S1. **SNP effects cross the whole genome at the heritability of 0.1. **Fig. S2.** SNP effects cross the whole genome at the heritability of 0.3. **Fig. S3.** SNPeffects cross the whole genome at the heritability of 0.5.

## Data Availability

The data and computing programs used in this manuscript are available from the corresponding author on request.

## Abbreviations

BLUP: Best linear unbiased prediction
EBV: Estimated breeding value
GEBV: Genomic estimated breeding value
GM: Genomic mating
GRM: Genomic relationship matrices
GS: Genomic selection
IBD: Identity by Decent
KPI: Key performance indicators
LD: Linkage disequilibrium
NRM: Numerator relationship matrix
OCS: Optimal contribution selection
QTL: Quantitative trait locus
ROH: Runs of homozyosity
SNP: Single nucleotide polymorphism
