# Supplementary material for: Genetic gain and inbreeding from simulation of different genomic mating schemes for pig improvement
Source: J Anim Sci Biotechnol. 2023 Jun 13;14:87. doi: 10.1186/s40104-023-00872-x (PMC10262571; doi:10.1186/s40104-023-00872-x)
Supplement: Supplementary file 1 — Additional file 1: Fig. S1. SNP effects cross the whole genome at the heritability of 0.1. Fig. S2. SNP effects cross the whole genome at the heritability of 0.3. Fig. S3. SNPeffects cross the whole genome at the heritability of 0.5. [file 40104_2023_872_MOESM1_ESM.docx]

Supplementary Information


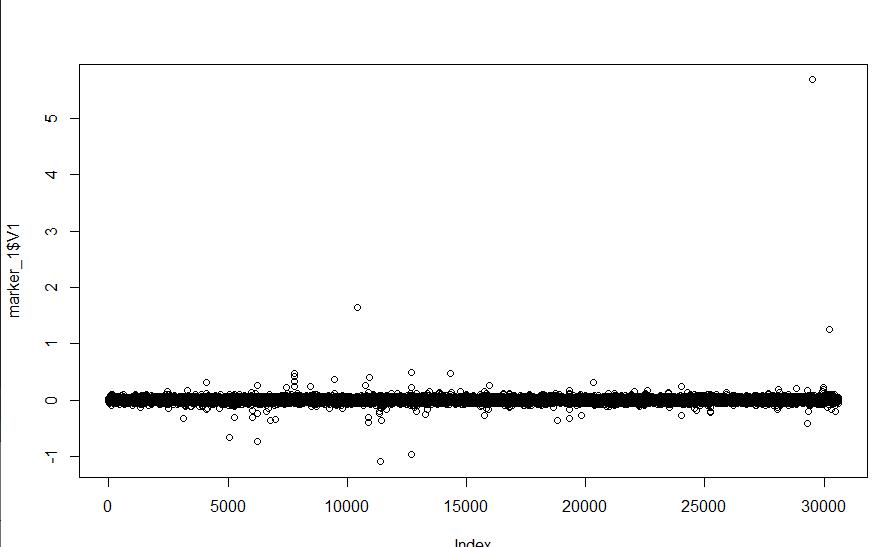


**Fig. S1** SNP effects cross the whole genome at the heritability of 0.1


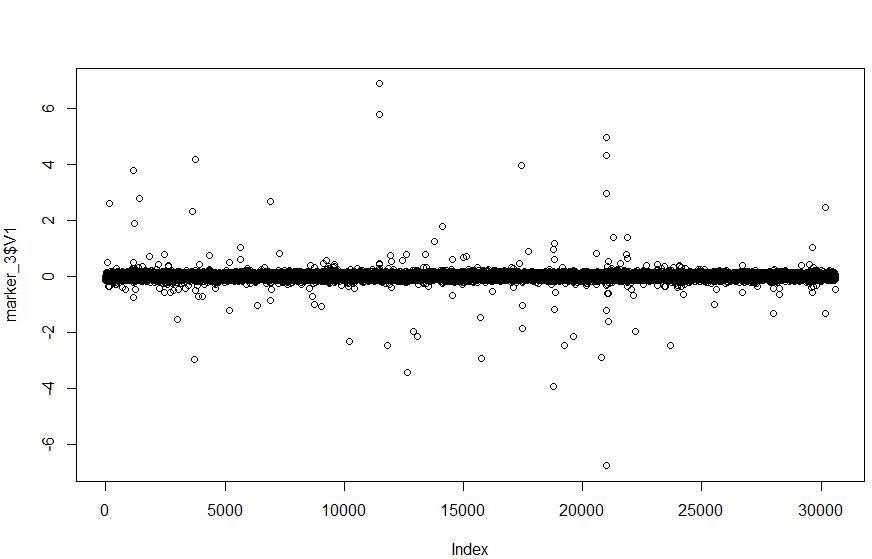


**Fig. S2** SNP effects cross the whole genome at the heritability of 0.3


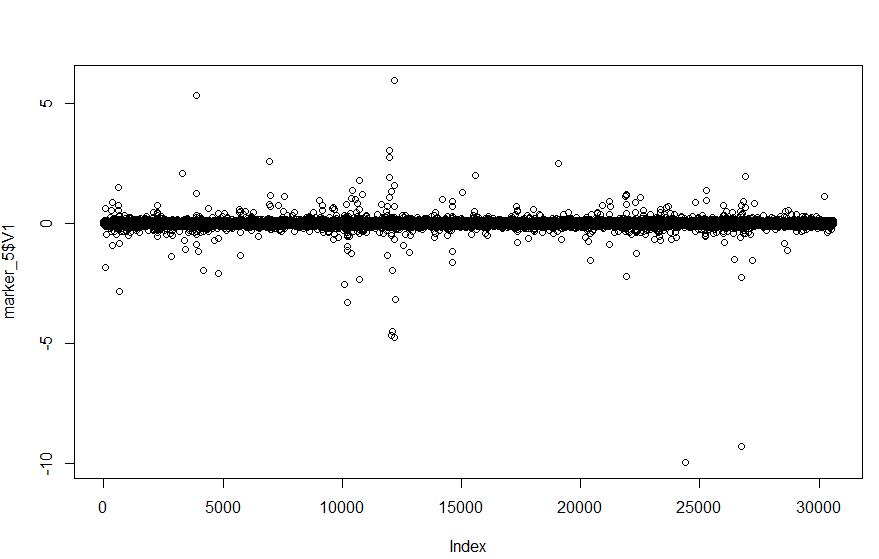


**Fig. S3** SNP effects cross the whole genome at the heritability of 0.5
